# Complexity of line-*seru* conversion for different scheduling rules and two improved exact algorithms for the multi-objective optimization

**DOI:** 10.1186/s40064-016-2445-5

**Published:** 2016-06-21

**Authors:** Yang Yu, Sihan Wang, Jiafu Tang, Ikou Kaku, Wei Sun

**Affiliations:** Institute of Systems Engineering, Northeastern University, Shenyang, 110819 People’s Republic of China; College of Management Science and Engineering, Dongbei University of Finance and Economics, Shahekou, Dalian, 116026 People’s Republic of China; Faculty of Environmental and Information Studies, Tokyo City University, Ushikubonishi 3-3-1, Yokohama, 224-8551 Japan; Business School, Liaoning University, Shenyang, 110316 People’s Republic of China

**Keywords:** Manufacturing, Scheduling rule, Time complexity, Space complexity, Exact algorithm

## Abstract

Productivity can be greatly improved by converting the traditional assembly line to a *seru* system, especially in the business environment with short product life cycles, uncertain product types and fluctuating production volumes. Line-*seru* conversion includes two decision processes, i.e., *seru* formation and *seru* load. For simplicity, however, previous studies focus on the *seru* formation with a given scheduling rule in *seru* load. We select ten scheduling rules usually used in *seru* load to investigate the influence of different scheduling rules on the performance of line-*seru* conversion. Moreover, we clarify the complexities of line-*seru* conversion for ten different scheduling rules from the theoretical perspective. In addition, multi-objective decisions are often used in line-*seru* conversion. To obtain Pareto-optimal solutions of multi-objective line-*seru* conversion, we develop two improved exact algorithms based on reducing time complexity and space complexity respectively. Compared with the enumeration based on non-dominated sorting to solve multi-objective problem, the two improved exact algorithms saves computation time greatly. Several numerical simulation experiments are performed to show the performance improvement brought by the two proposed exact algorithms.

## Background

The *seru* production, conceived at Sony, is an innovation of assembly system used widely in the Japanese electronics industry and recognized a new production patten. *Seru* is a manufacturing organization (an assembly unit) that consists of simple equipment and one or several worker(s) that are dedicated to one or several product(s). In *seru*, worker(s) must be multi-skilled operators, i.e., workers can operate the most or all processes of production.

To compete in a turbulent market, in 1992, several mini-assembly units were created in one of Sony’s video-camera factories for an 8-mm CCD-TR55 video-camera, after dismantling a long assembly conveyor line. As did the original conveyor line, each mini-assembly unit produced the entire product. In 1994, Tatsuyoshi Kon, a former Sony staff, called this mini-assembly organization as *seru*, a Japanese word for cellular organism. *Seru* is similar to assembly cells, a widely adopted assembly system in western industries. Equipment, however, is less important for *seru*. As a human-centered assembly system, *seru* is an old-fashioned workshop where craftsperson, including jack-of-all-trades workers, assembles an entire product from-start-to-finish by her- or himself. This mini-assembly organization is regarded as an ideal combination of lean and agile production paradigms. By adopting *seru* production, Canon and Sony reduced 720,000 and 710,000 m^2^ of floor space, respectively (Stecke et al. 2012). Cost can also be reduced largely by using *seru* systems. After adopting *seru* systems, Canon’s costs were reduced significantly, by 55 billion yen in 2003, and by a total of 230 billion yen from 1998 to 2003. As a result, Canon emerged as a leading electronics maker. Its average productivity is higher than that of Toyota (Yin et al. 2008). Other benefits from *seru* systems include the reductions of throughput time, setup time, required labor hours, WIP inventories, and finished-product inventories. Some amazing cases related to the reductions in throughput time and required labor hours, the throughput time was reduced by 53 % at Sony Kohda and 35,976 required workers, equal to 25 % of Canon’s previous total workforce, have been saved.

There are three types of *seru*: divisional *seru*, rotating *seru*, and *yatai*. A divisional *seru* is a short line staffed with several partially cross-trained workers. Tasks within a divisional *seru* are divided into different sections. Each section is operated by one or more workers. Workers staffed within rotating *seru* or *yatais* are completely cross-trained. A rotating *seru* is often organized in a U-shaped layout with several workers. Each worker assembles an entire product from-start-to-finish without disruption. A *yatai* is the *seru* with a single worker who does all operational and managerial tasks. An NEC (Nippon Electric Company in Japan) completely cross-trained worker can assemble a word processor of 120 components in 18 min (Shinohara 1995; Stecke et al. 2012). In this research, the *serus* are rotating *serus* or *yatai*. A detailed introduction of *seru* system and its managerial mechanism can be found in Yin et al. (2008), Liu et al. (2010) and Stecke et al. (2012).

Due to the merit of *seru* production, many companies converted assembly line into *seru* system to increase the productivity. The line-*seru* (or line-cell) conversion was used widely in the Japanese electronics industry (Isa and Tsuru 1999; Miyake 2006; Sakazume 2005, 2012; Shinobu 2009; Yoshimoto 2003). Its essence is to convert traditional conveyor assembly line to a *seru* system in which one (or multiple) worker performs the most of all tasks the *seru*. The total productivity of manufacturers may be increased dramatically by line-*seru* conversion (Johnson 2005; Kaku et al. 2009; Stecke et al. 2012; Yin et al. 2008). Liu et al. (2014) proposed an implementation framework and process for converting the assembly line into a *seru* system.

**Fig. 1:**
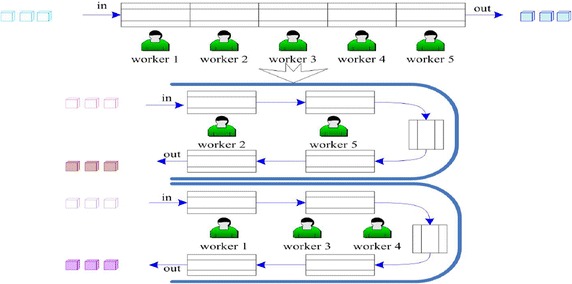
An example of converting assembly line to a pure *seru* system (Yu et al. 2012)

The first issue of line-*seru* conversion is to establish the mathematical model. Such technical and decision making problems had been defined as line-*seru* conversion problems (Kaku et al. 2009). Kaku et al. (2009) considered three types of systems including a pure *seru* system (as shown in Fig. 1, where two *serus* are constructed, i.e., workers 2 and 5 in *seru* 1 and workers 1, 3 and 4 in *seru* 2), a pure assembly line and a hybrid system with *serus* and line. The pure *seru* system is very simple and a special case of all other *seru* assembly systems. The results obtained for pure *seru* system models not only provide insights into the pure *seru* system environment, they also provide a basis for heuristics that are applicable to more complicated assembly *seru* system environments. Therefore, many literatures focused on converting the assembly line to a pure *seru* system, such as Yu et al. (2012, 2013, 2014). Also, the research considers the assembly line is converted to a pure *seru* system.

Another key of line-*seru* conversion is to evaluate the performance improvement created by the conversion. Kaku et al. (2009) used total throughput time (TTPT) and total labor hours (TLH) to evaluate the performance improvement created by line-*seru* conversion. Kaku et al. (2009) and Yu et al. (2012) investigated the operational influence factors to TTPT and TLH. They summarized several managerial insights that could be used to improve the performances of TTPT and TLH through line-*seru* conversion. Yu et al. (2013) evaluated the performance improvement from the perspective of manpower reduction. They established the line-*seru* conversion model towards reducing worker(s) and proposed an exact algorithm.

Also, the decision problem in line-*seru* conversion is widespread concerned. In fact, line-*seru* conversion includes two decision problems, i.e., *seru* formation and *seru* load (Yu et al. 2012, 2013, 2014). Most of previous researches focused on *seru* formation. Yu et al. (2012) investigated how to format *serus* to improve the performances of TTPT and TLH. Yu et al. (2013) clarified the complexity of *seru* formation towards reducing workers. Yu et al. (2014) revealed the mathematical characteristics of *seru* formation such as solution space, complexity and non-convex properties. Regarding *seru* load, most researches used given scheduling rule to assign product batches to *serus*, because *seru* load is NP-hard. Yu et al. (2012, 2013, 2014) used the FCFS (First Come First Severed) rule to dispatch product batches into *serus*. Therefore, the *seru* load should be investigated.

This paper, originally motivated by line-*seru* conversion applications of Sony and Canon, has two purposes. First, we demonstrate the influence of ten different scheduling rules usually used in *seru* load on the performance of line-*seru* conversion. Subsequently, we clarify exactly the complexities of *seru* load and line-*seru* conversion for the ten scheduling rules. Second, to obtain Pareto-optimal solutions for the large-scale instances of line-*seru* conversion, we propose two improved exact algorithms by decreasing time complexity and space complexity respectively.

The remainder of this study is organized as follows. The bi-objective model of converting the assembly line into a pure *seru* system with minimizing TTPT and TLH is given in the second section. The third section illustrates the influence of ten scheduling rules on the TTPT and TLH performances of *seru* system. The forth section clarifies exactly the complexities of *seru* load and line-*seru* conversion for ten scheduling rules. In the fifth section, two exact algorithms are developed based on reducing time complexity and space complexity. Several examples to illustrate the performance of the two proposed algorithms are given in the sixth section. In the last section conclusions and future research are given. All theorem proofs can be found in the “Appendix”.

## Multi-objective model of converting the assembly line into a pure *seru* system

### Assumption

The following assumptions are considered in this study.

1. The types and batches of products to be processed are known in advance. There are *N* product types that are divided into *M* product batches. Each batch contains a single product type.
2. The assembly tasks within a *seru* are manual so need only simple and cheap equipment and the cost of duplicating equipment is ignored (Stecke et al. 2012; Yu et al. 2012).
3. A product batch is assembled entirely within a *seru*.
4. All product types have the same assembly tasks. If a task is not used in a product, then we assume the task time for the product was zero.
5. In the assembly line, each task (or station) is in the charge of a single worker. That means that a worker only performs a single assembly task in the assembly line. Therefore, the total number of tasks in the line equals *W*.
6. The assembly tasks within each *seru* are the same as the ones within the line. A *seru* worker needs to perform all assembly tasks, assembles an entire product from-start-to-finish, and there is no disruption or delay between adjacent tasks.

### Indices

*i*: Index of workers (*i* = 1,2,…,*w*). *w* is the total number of workers in an assembly line.
*j*: Index of *serus* (*j* = 1,2,…,*J*). *J* is the total number of *serus* in a *seru* system.
*n*: Index of product types (*n* = 1, 2,…, *N*). *N* is the total number of product types.
*m*: Index of product batches (*m* = 1, 2,…, *M*). *M* is the total number of product batches.
*k*: Index of the sequence of product batches in a *seru* (*k* = 1, 2,…, *M*).
*q*: Index of sub-sets of all the feasible *seru* systems (*q* = 1, 2,…, *Q*). *Q* is the total number of sub-sets.

### Parameters

$$Vmn = \left\{ \begin{array}{ll} 1,&\quad{\text{if product type of product batch }}m{\text{ is }}n \hfill \\ 0,&\quad{\text{otherwise }} \hfill \\ \end{array} \right..$$

*B*_*m*_: Size of product batch *m*.
*T*_*n*_: Cycle time of product type *n* in the assembly line.
*SL*_*n*_: setup time of product type *n* in the assembly line.
*SCP*_*n*_: Setup time of product type *n* in a *seru*.
*T*_*mj*_: Average task time of *seru j* assembling product batch *m*.
*η*_*i*_: Upper bound on the number of tasks for worker *i* in a *seru*. If the number of tasks assigned to worker *i* is more than *η*_*i*_, worker *i’s* average task time within a *seru* will be longer than her or his task time within the original assembly line.
*ε*_*i*_: Worker *i’s* coefficient of influencing level of doing multiple assembly tasks.
*β*_*ni*_: Skill level of worker *i* for each task of product type *n*.

### Decision variables

$$Xij = \left\{ {\begin{array}{*{20}l} {1,} \hfill & {{\text{if}}\;{\text{worker}}\;i\;{\text{is}}\;{\text{assigned}}\;{\text{to}}\;{\text{seru}}\;j} \hfill \\ {0,} \hfill & {\text{otherwise }} \hfill \\ \end{array} } \right..$$

$$Zmjk = \left\{ {\begin{array}{*{20}l} {1,} \hfill & {{\text{if}}\;{\text{product}}\;{\text{batch}}\;m\;{\text{is}}\;{\text{assigned}}\;{\text{to}}\;{\text{seru}}\;j\;{\text{in}}\;{\text{sequence}}\;k} \hfill \\ {0,} \hfill & {\text{otherwise}} \hfill \\ \end{array} } \right..$$

### Variables

*C*_*i*_: Coefficient of variation of worker *i’s* increased task time after line-*seru* conversion, i.e., from a specialist to a completely cross-trained worker. if the number of worker *i’s* tasks within a *seru* is over her or his upper bound *η*_*i*_, i.e., *w* > *η*_*i*_, then the worker will cost more average task time than her or his task time within the original assembly line. *c*_*i*_ is given in Eq. (1).
*TC*_*m*_: Assembly task time of product batch *m* per station in a *seru*. In a *seru*, the task time of product type *n* is calculated by the average task time of workers in the *seru*. *TC*_*m*_ is represented as Eq. (2).
*FCB*_*m*_: Begin time of product batch *m* in a *seru*. There is no waiting time between two product batches so that *FCB*_*m*_ is the aggregation of flow time and setup time of the product batches processed prior to product batch *m* in the same *seru*. *FCB*_*m*_ is represented as Eq. (3).
*SC*_*m*_: Setup time of product batch *m* in a *seru*. Setup time is considered when two different types of products are processed consecutively; otherwise the setup time is zero. For example, in Eq. (4), two adjacent assembled products in a *seru* are expressed as *m* and *m*′. If the product type of *m* is different with that of *m*′, i.e., *V*_*mn*_ = 1, $V_{{m^{{\prime }} n}} = 0$, and then the setup time of batch *m* is *SCP*_*n*_*V*_*mn*_. However, if the product types of *m* and *m*′ are identical, i.e., $V_{mn} = V_{{m^{{\prime }} n}} = 1$, and then the setup time of batch *m* is 0.
*FC*_*m*_: Flow time of product batch *m* in a *seru*. *FC*_*m*_ is represented as Eq. (5).

1 $$C_{i} = \left\{ {\begin{array}{*{20}l} {\begin{array}{*{20}l} {1 + \varepsilon_{i} (W - \eta_{i} ),} \hfill & {W > \eta_{i} } \hfill \\ {1,} \hfill & {W \le \eta_{i} } \hfill \\ \end{array} } \hfill & {\forall i} \hfill \\ \end{array} } \right.$$

2 $$TC_{m} = \frac{{\sum\nolimits_{n = 1}^{N} {\sum\nolimits_{i = 1}^{W} {\sum\nolimits_{j = 1}^{J} {\sum\nolimits_{k = 1}^{M} {V_{mn} T_{n} \beta_{ni} C_{i} X_{ij} Z_{mjk} } } } } }}{{\sum\nolimits_{i = 1}^{W} {\sum\nolimits_{j = 1}^{J} {\sum\nolimits_{k = 1}^{M} {X_{ij} Z_{mjk} } } } }}$$

3 $$FCB_{m} = \sum\limits_{s = 1}^{m - 1} {\sum\limits_{j = 1}^{J} {\sum\limits_{k = 1}^{m} {(FC_{s} + SC_{s} )Z_{mjk} Z_{sj(k - 1)} } } }$$

4 $$SC_{m} = \left\{ {\begin{array}{*{20}c} {\begin{array}{*{20}l} {SCP_{n} V_{mn} ,} \hfill & {V_{mn} = 1,\;V_{{m^{{\prime }} n}} = 0} \hfill \\ {0,} \hfill & {V_{mn} = V_{{m^{{\prime }} n}} = 1} \hfill \\ \end{array} } & {(m^{{\prime }} |Z_{mjk} = 1,\;Z_{{m^{{\prime }} j(k - 1)}} = 1,\quad \forall j,k)} \\ \end{array} } \right.$$

5 $$FC_{m} = \frac{{B_{m} TC_{m} W}}{{\sum\nolimits_{i = 1}^{W} {\sum\nolimits_{j = 1}^{J} {\sum\nolimits_{k = 1}^{M} {X_{ij} Z_{mjk} } } } }}$$

### TTPT and TLH of the *seru* system

The total throughput time (TTPT) and the total labor hours (TLH) of the *seru* system are expressed as follows.

6 $${\text{TTPT}}\;{\text{of}}\;seru\;{\text{system}} = \mathop {\text{Max}}\limits_{m} (FCB_{m} + FC_{m} + SC_{m} )$$

7 $${\text{TLH}}\;{\text{of}}\;seru\;{\text{system}} = \sum\limits_{m = 1}^{M} {\sum\limits_{i = 1}^{W} {\left( {\sum\limits_{j = 1}^{J} {\sum\limits_{k = 1}^{M} {FC_{m} } } X_{ij} Z_{mjk} } \right)} }$$

TTPT of *seru* system is the completion time of the last completed product batch. TLH of *seru* system is the cumulative working time of all workers in the *seru* system. Given product batches, the *seru* systems should have shorter TTPT and TLH than the line.

### Formulation of bi-objective line-*seru* conversion with minimizing TTPT and TLH

The mathematical model is formulated as Eqs. (8)–(13).

Objective functions:

8 $${\text{Min}}\;{\text{TTPT}}\;{\text{of}}\;seru\;{\text{system}}$$

9 $${\text{Min}}\;{\text{TLH}}\;{\text{of}}\;seru\;{\text{system}}$$

Subject to:

10 $$1 \le \sum\limits_{i = 1}^{W} {X_{ij} } \le W,\quad \forall j$$

11 $$\sum\limits_{j = 1}^{J} {X_{ij} } = 1,\quad \forall i$$

12 $$\sum\limits_{j = 1}^{J} {\sum\limits_{k = 1}^{M} {Z_{mjk} } } = 1,\quad \forall m$$

13 $$\sum\limits_{m = 1}^{M} {\sum\limits_{k = 1}^{M} {Z_{mjk} } } = 0,\quad \left(\forall j|\sum\limits_{i = 1}^{W} {X_{ij} } = 0\right)$$

where Eq. (8) minimizes the total throughput time (TTPT). Equation (9) minimizes the total labor hours (TLH). Equation (10) is the number constraint that the number of workers within a *seru* must be in the interval of [1, *W*]. Equation (11) is the worker assignment rule, i.e., each worker should be assigned to one and only one *seru*. Equation (12) is the product batch assignment rule, i.e., each batch should be assigned to one and only one *seru*. Equation (13) is the rule of assigning constraint, i.e., a product must be assigned to a *seru* in which at least one worker is assigned. In other words, for a *seru* without any worker, i.e., $\forall j|\sum\nolimits_{i = 1}^{W} {X_{ij} } = 0$, any batch cannot be assigned into the *seru*, i.e., $\sum\nolimits_{m = 1}^{M} {\sum\nolimits_{k = 1}^{M} {Z_{mjk} } } = 0$.

## Influence of the scheduling rules on line-*seru* conversion

Line-*seru* conversion includes *seru* formation and *seru* load (Yu et al. 2012). *Seru* load determines which product batches are dispatched to the *serus* formed in *seru* formation. *Seru* formation determines how many *serus* to be constructed and how to assign the workers into *serus*. A detailed introduction of *seru* formation can be found in Yu et al. (2012, 2013, 2014).

### **Property 1**

*Given a seru formation, without a given scheduling ruleseru load is NP*-*hard*.

### **Explanation**

Without a given scheduling rule, in *seru* load, each product batch can be assigned into any *seru* in the given *seru* formation. Therefore, *seru* load is an assignment and NP-hard problem.

Thus, for simplicity, earlier researches (Kaku et al. 2009; Yu et al. 2012, 2013, 2014) fixed the scheduling rules on *seru* load such as FCFS and SPT.

However, the different scheduling rules produce different performances or complexity of a system (Chuen and Robert 1993; Grabot and Geneste 1994; Holthaus and Rajendran 1997; Amirghasemi and Zamani 2014; Nurre and Sharkey 2014; Xu et al. 2015; Zeng et al. 2015). The comparative analysis of scheduling rules in some specific industrial environments can be found in Rajendran and Holthaus (1999), Kizil et al. (2006), Chiang and Fu (2007), Akturk (2011), and Li et al. (2015).

To investigate the influence of scheduling rules on the performance of line-*seru* conversion, we used a total of ten scheduling rules of *seru* load. The ten scheduling rules are selected from *Seru* production applications of Sony and Canon, because the ten rules are usually used in *Seru* production. The ten scheduling rules used in the paper are defined in detailed as follows.

To illustrate clearly that TTPT and TLH of line-*seru* conversion are influenced by the different scheduling rules, we used 5 product batches and 2 *serus*, where worker 1 in *seru* 1 and worker 2 in *seru* 2. The data of task time of 5 batches on 2 *seru* is shown in Table 1. The data of the earliest due date (*EDD*) of 5 batches is shown in Table 2.

**Table 1:** Task time of five batches on 2 *serus*

*Serus*\batches	1	2	3	4	5
*Seru*1 {1}	2	4	3	4	3
*Seru*2 {2}	1	6	4	2	4

*Seru*1 {1} and *Seru*2 {2} mean worker 1 in *seru* 1 and worker 2 in *seru* 2. The *seru* sequence is {1}-{2}

**Table 2:** Earliest due date of 5 batches

Batches	1	2	3	4	5
EDD	6	9	10	5	8

### **First come first served (FCFS)**

**Fig. 2:**
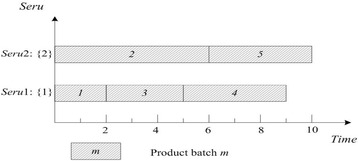
Result of FCFS rule on *seru* load with the *seru* sequence of {1}–{2}

This rule is often used as a bench-mark. FCFS of *seru* load is described as following: an arriving product batch is assigned to the empty *seru* with the smallest *seru* number. If all *serus* are occupied, the product batch is assigned to the *seru* with the earliest completion time. Result with FCFS on *seru* load shows in Fig. 2, where TTPT is 10 and TLH is 19.

### **Last come first served (LCFS)**

**Fig. 3:**
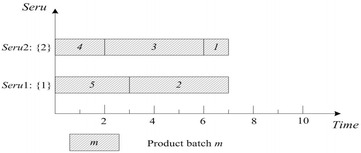
Result of LCFS rule on *seru* load with the *seru* sequence of {1}–{2}

LCFS of *seru* load is described as following: the last arriving product batch is assigned to the empty *seru* with the smallest *seru* number. If all *serus* are occupied, the product batch is assigned to the *seru* with the earliest completion time. Result with LCFS on *seru* load shows in Fig. 3, where TTPT is 7 and TLH is 14.

### **Shortest processing time (SPT)**

**Fig. 4:**
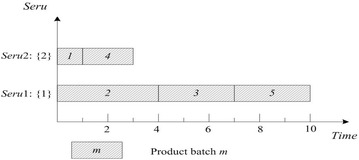
Result of SPT rule on *seru* load with the *seru* sequence of {1}–{2}

This rule is perhaps the most commonly used rule for job shop scheduling. SPT of *seru* load is described as following: an arriving product batch is assigned to the *seru* with the shortest processing time for it. The shortest processing time (SPT) of batch *m* is $\mathop {\hbox{min} }\nolimits_{j = 1}^{J} (Tmj)$, e.g., SPTs of batches 1 and 2 are 1 and 4 respectively. Result with SPT on *seru* load shows in Fig. 4, where TTPT is 10 and TLH is 13.

### **Earliest completion time (ECT)**

**Fig. 5:**
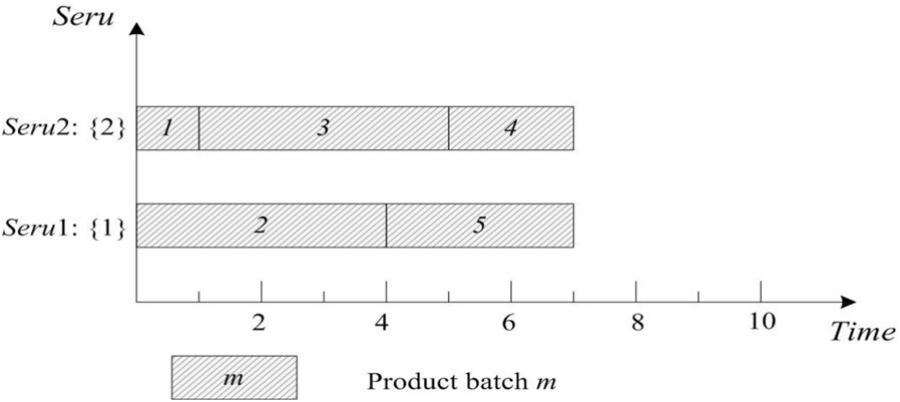
Result of ECT rule on *seru* load with the *seru* sequence of {1}–{2}

As shown in Fig. 4, SPT rule on *seru* load may cause the imbalance among *serus*. For example, the difference between the two *serus* in Fig. 4 is 7 = 10-3. Therefore, Yu et al. (2012) proposed that the balance among *serus* should be considered in *seru* load. ECT of *seru* load is described as following: an arriving product batch is assigned to the *seru* with the earliest completion time of finishing the batch. Result with ECT on *seru* load shows in Fig. 5, where TTPT is 7, TLH is 14, and the difference between the two *serus* is 0.

### **Earliest due-date first (EDD)**

**Fig. 6:**
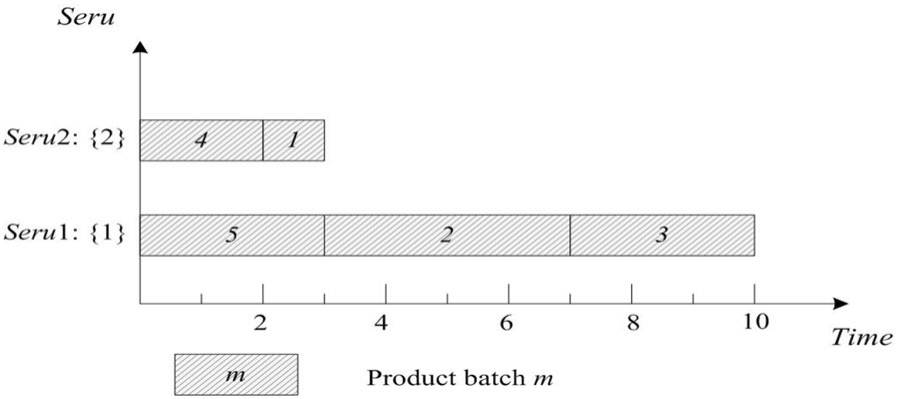
Result of EDD rule on *seru* load with the *seru* sequence of {1}–{2}

This rule is often used in industries for its simplicity of implementation. EDD of *seru* load is described as following: the product batch with the earliest due-date is selected and assigned to the *seru* with the shortest processing time for the batch. Result with EDD on *seru* load shows in Fig. 6, where TTPT is 10 and TLH is 13.

### **Modified earliest due-date first (MEDD)**

**Fig. 7:**
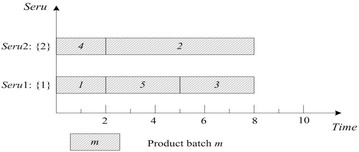
Result of MEDD rule on *seru* load with the *seru* sequence of {1}–{2}

MEDD of *seru* load is described as following: the product batch with the earliest due-date is selected and assigned to the *seru* with the earliest completion time of finishing the batch. Result with MEDD on *seru* load shows in Fig. 7, where TTPT is 8 and TLH is 16.

### **Minimal Shortest Processing Time first (MSPT)**

**Fig. 8:**
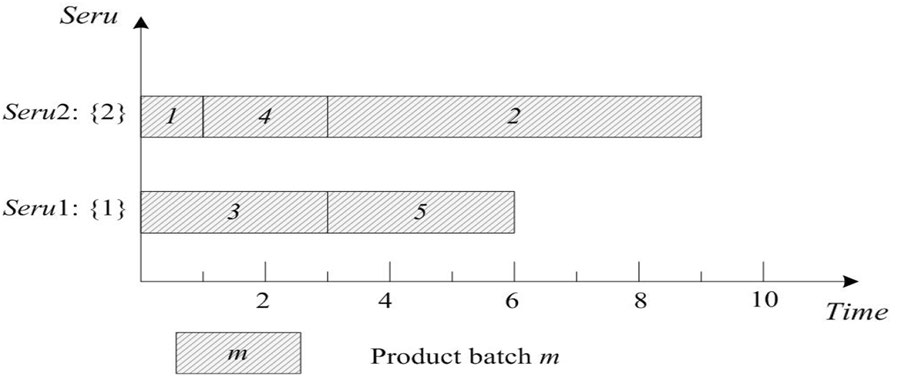
Result of MSPT rule on *seru* load with the *seru* sequence of {1}–{2}

MSPT of *seru* load is described as following: the product batch with the minimal shortest processing time is selected and assigned to the *seru* with the shortest processing time for it. MSPT is the minimal SPT of all batches, i.e., $\mathop {\hbox{min} }\nolimits_{m = 1}^{M} \mathop {\hbox{min} }\nolimits_{j = 1}^{J} (Tmj)$. For example, MSPT of Table 1 is 1. Result with MSPT on *seru* load shows in Fig. 8, where TTPT is 10 and TLH is 13.

### **Modified minimal shortest processing time first (MMSPT)**

**Fig. 9:**
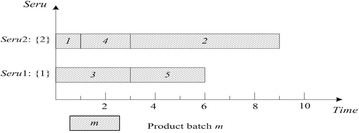
Result of MMSPT rule on *seru* load with the *seru* sequence of {1}–{2}

MMSPT of *seru* load is described as following: the product batch with the minimal shortest processing time is selected and assigned to the *seru* with the earliest completion time of finishing the batch. Result with MMSPT on *seru* load shows in Fig. 9, where TTPT is 9 and TLH is 15.

### **Longest shortest processing time first (LSPT)**

**Fig. 10:**
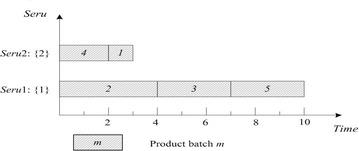
Result of LSPT rule on *seru* load with the *seru* sequence of {1}–{2}

LSPT of *seru* load is described as following: the product batch with the longest shortest processing time is selected and assigned to the *seru* with the shortest processing time for it. LSPT is the longest SPT of all batches, i.e., $\mathop {\hbox{max} }\nolimits_{m = 1}^{M} \mathop {\hbox{min} }\nolimits_{j = 1}^{J} (Tmj)$. For example, LSPT of Table 1 is 4. Result with LSPT on *seru* load shows in Fig. 10, where TTPT is 10 and TLH is 13.

### **Modified longest shortest processing time first (MLSPT)**

**Fig. 11:**
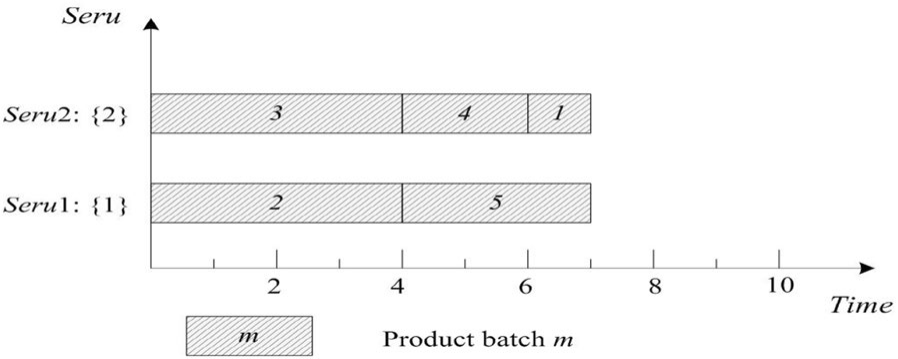
Result of MLSPT rule on *seru* load with the *seru* sequence of {1}–{2}

MLSPT of *seru* load is described as following: the product batch with the longest shortest processing time is selected and assigned to the *seru* with the earliest completion time of finishing the batch. Result with MLSPT on *seru* load shows in Fig. 11, where TTPT is 7 and TLH is 14.

Based on Figs. 2, 3, 4, 5, 6, 7, 8, 9, 10 and 11, we can obtain Table 3. Table 3 shows that the different scheduling rules on *seru* load cause different TTPTs and TLHs of the converted *seru* system even though the *seru* formation is identical.

**Table 3:** Result of 10 scheduling rules used in *seru* load with the same *seru* formation

Scheduling rules	FCFS	LCFS	SPT	ECT	EDD	MEDD	MSPT	MMSPT	LSPT	MLSPT
TTPT	10	7	10	7	10	8	9	9	10	7
TLH	19	14	13	14	13	16	15	15	13	14

From Table 3, we can see that scheduling rules have a significant effect on the performance of converted *seru* system with the same *seru* formation. For example, the best and worst *TTPT* in the ten scheduling rules are 7 and 10 respectively, and the best and worst *TLH* in the ten scheduling rules are 13 and 19. Therefore, the investigation on scheduling rules used in *seru* load is important for *Seru* production. Consequently, we clarify the complexities of solution spaces of *seru* load and line-*seru* conversion for the ten scheduling rules from the theoretical perspective. The clarification of complexity of solution space makes it possible to obtain the optimal solution or Pareto-optimal solutions of line-*seru* conversion.

## Complexity of *seru* load and line-*seru* conversion for the different scheduling rules

The line-*seru* conversion is a two-stage decision process, i.e., *seru* formation and *seru* load (Yu et al. 2012, 2014). Therefore, the complexity of line-*seru* conversion should be clarified by combining the complexity of *seru* formation with the complexity of *seru* load.

### Complexity of *seru* formation

*Seru* formation is the first step of line-*seru* conversion. Distinguished from the traditional manufacturing cell formation problems (Safaei and Tavakkoli-Moghaddam 2009; Wu et al. 2009), *seru* formation in line-*seru* conversion is to determine how many *serus* to be formed and how to assign workers into the *serus* (Yu et al. 2012). *Seru* formation is decided by decision variable *X*_*ij*_.

#### **Property 2**

*Seru formation of line*-*seru conversion is an instance of the unordered set partition problem and NP*-*hard*.

#### **Explanation**

*Seru* formation is to partition a conveyor line with *W* workers into pair-wise disjoint nonempty *serus*, and so *seru* formation is an instance of the unordered set partition problem. Set partitioning is a well-known NP-hard problem (Garey and Johnson 1979). The detailed proof can be found in Yu et al. (2013).

Since *seru* formation is an instance of the unordered set partition, the number of all the feasible solutions of *seru* formation can be expressed as:

14 $$F(W) = \sum\limits_{J = 1}^{W} {P (W ,J )}$$

where *P*(*W*, *J*) is the count of partitioning *W* workers in assembly line into *Jserus* and can be expressed as the Stirling numbers of the second kind (Rennie and Dobson 1969; Williamson 1985; Knopfmacher and Mays 2006; Klazar 2003).

## Complexities of *seru* load for the different scheduling rules

*Seru* load is the second step of line-*seru* conversion and is decided by decision variable *Z*_*mjk*_. It determines which product batches are dispatched to the *serus* formed in *seru* formation (Chen et al. 2013; Solimanpur and Elmi 2013).

According to Properties 1 and 2, line-*seru* conversion is a complex problem including two NP-hard problems (i.e., *seru* formation and *seru* load). For simplicity and without loss of generality, the scheduling rule in *seru* load is usually given. However, even given a scheduling rule in *seru* load, line-*seru* conversion is still an NP-hard problem because *seru* formation is NP-hard.

Different scheduling rules produce different performances or complexity of line-*seru* conversion. Up to now, the influences of scheduling rules to line-*seru* conversion are not investigated yet. Therefore, one objective of this study is to clarify the influence of different scheduling rules to complexity of line-*seru* conversion. Since the complexity of *seru* formation is independent of scheduling rule, we focus on clarifying the influence of different scheduling rules to complexity of *seru* load.

Gven a *seru* formation, the number of solutions (*S*) of *seru* load can be expressed by the number of *serus* (*J*).

### **Theorem 1**

*Given a seru formation with J serus, without given a scheduling rule, S* =  *J*^*M*^.

### *Proof*

See Proof of Theorem 1 in “Appendix”.

As described in Property 1 and Theorem 1, *seru* load is NP-hard and has *J*^*M*^ feasible solutions. For simplicity, therefore, earlier researches used the typical scheduling rules such as FCFS and SPT.

In addition, the number of solutions (*S*) of *seru* load varies with the scheduling rules. For example, for the line with two workers labeled 1 and 2, there are two solutions of *seru* formation, i.e., {{1,2}} and {{1},{2}}. For the latter solution of {{1},{2}}, there are two *seru* sequences, i.e., {1}–{2} and {2}–{1}.

**Fig. 12:**
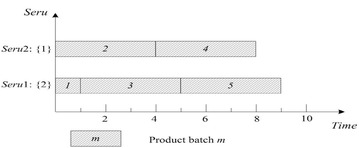
Result with FCFS rule on *seru* load with the *seru* sequence of {2}–{1}

For the 5 product batches in Table 1, there are two results of *seru* load with FCFS. The result of {1}–{2} is shown in Fig. 2 and the result of {2}–{1} is shown in Fig. 12. This means the *seru* sequence does influence the result of *seru* load with FCFS.

**Fig. 13:**
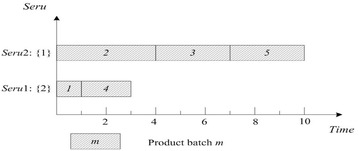
Result with SPT rule on *seru* load with the *seru* sequence of {2}–{1}

However, for the 5 product batches in Table 1, there is only one result of *seru* load with SPT. For the *seru* sequence of {1}–{2}, the result of *seru* load with SPT is shown in Fig. 4. For the *seru* sequence of {2}–{1}, the result of *seru* load with SPT is shown in Fig. 13. By comparing Figs. 4 and 13, we can easily observe that the two results are identical. This is because regardless of the *seru* sequence, using SPT an arriving batch is always assigned to the *seru* with the shortest processing time for it. This means the *seru* sequence does not influence the result of *seru* load with SPT.

Therefore, the ten scheduling rules are divided into two classes: (1) scheduling rules related to *seru* sequence (*SRRSS*); and (2) scheduling rules unrelated to *seru* sequence (*SRUSS*). A *SRRSS* rule is the one with which the *seru* load result is influenced by the *seru* sequence. However, a *SRUSS* rule means that the *seru* load result is independent of the *seru* sequence using the rule. In the ten scheduling rules, FCFS and LCFS belong to *SRRSS*, but the other eight scheduling rules belong to *SRUSS*. Thus, we clarify the complexity of *seru* load with the ten scheduling rules from the two classes.

Complexity (*S*) of *seru* load with *SRRSS* is clarified in Theorems 2–3. Complexity (*S*) of *seru* load with *SRUSS* is clarified in Theorem 4.

### **Theorem 2**

*Given a seru formation with J serus, if seru load uses a SRRSS and M* ≥ *J, S* = *J!* = $P_{J}^{J}$.

### *Proof*

See Proof of Theorem 2 in “Appendix”.

### **Theorem 3**

*Given a seru formation with J serus, if seru load uses a SRRSS and M* < *J, S* = $C_{J}^{M} P_{M}^{M} = P_{J}^{M}$.

### *Proof*

See Proof of Theorem 3 in “Appendix”.

### **Theorem 4**

*Given a seru formation with J serus, if seru load uses a SRUSS, S* = *1*.

### *Proof*

See Proof of Theorem 4 in “Appendix”.

Subsequently, the complexity (*T*(*W*)) of line-*seru* conversion with the different scheduling rules can be clarified by combining the complexity of *seru* formation (*F*(*W*)) with the complexity of *seru* load (*S*).

### Complexities of line-*seru* conversion with the different scheduling rules

The complexities (*T*(*W*)) of line-*seru* conversion with the different scheduling rules are summarized in Table 4.

**Table 4:** Complexities (*T*(*W*)) of line-*seru* conversion with the different scheduling rules

Scheduling on *seru* load	*T*(*W*)	Explanation
Without given a scheduling	$\sum\limits_{J = 1}^{W} {P (W ,J )} *(J^{M} )$	Combine Eq. (14) with Theorem 1
A *SRRSS* and *M* ≥ *J*	$\sum\limits_{J = 1}^{W} {P (W ,J )} *(P_{J}^{J} )$	Combine Eq. (14) with Theorem 2
A *SRRSS* and *M* < *J*	$\sum\limits_{J = 1}^{W} {P (W ,J )} *(P_{J}^{M} )$	Combine Eq. (14) with Theorem 3
A *SRUSS*	$\sum\limits_{J = 1}^{W} {P (W ,J )}$	Combine Eq. (14) with Theorem 4

The clarification of complexity of solution space makes it possible to obtain the optimal solution or Pareto-optimal solutions of line-*seru* conversion.

## Two improved exact approaches for multi-objective line-*seru* conversion

Multi-objective decisions are often used in line-*seru* conversion (Kaku et al. 2009; Yu et al. 2013, 2014). However, multi-objective optimization is more difficult to solve than single-objective optimization (Ebrahimipour et al. 2015). Enumeration algorithm based on non-dominated sorting (Deb et al. 2002) for multi-objective line-*seru* conversion is described as follows.
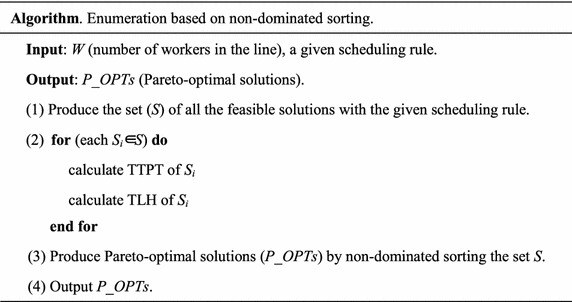


Step (1) is to produce all the feasible solutions (*N*) with the given scheduling rule. Both time complexity and space complexity are *O*(*N*).

Step (2) is to calculate TTPT and TLH of each feasible solution. Time complexity is *O*(*N*), but space complexity is *O*(4*N*) for storing *seru* sequence, TTPT, TLH and the batches assigned in each *seru*.

Step (3) is to obtain exact Pareto-optimal solutions by non-dominated sorting of Deb et al. (2002). Time complexity of non-dominated sorting is *O*(2*N*^2^), where 2 is the number of objectives. Space complexity is *O*(*N*).

Therefore, time complexity and space complexity of the enumeration algorithm are *O*(2*N*^2^) and *O*(4*N*) respectively.

However, because of the higher time complexity (i.e., *O*(2*N*^2^)), the enumeration cannot obtain the Pareto-optimal solutions for the instances with more than 6 workers using a *SRRSS*. We develop two improved exact algorithms for the large-scale instances by decreasing time complexity and space complexity respectively.

### The improved exact algorithm by decreasing time complexity

When the solutions attending non-dominated sorting are reduced by *R*, the time complexity will be improved by $\left( {1 - \frac{{(N - R)^{2} }}{{(N)^{2} }}*100\;\% } \right)$.

Therefore, we consider to cut off the solutions dominated by the certain Pareto-optimal solution(s) before running non-dominated sorting algorithm. The certain solutions are defined in Definitions 1 and 2.

#### **Definition 1**

mTTPT is the Pareto-optimal solution with the minimal TTPT.

#### **Definition 2**

mTLH is the Pareto-optimal solution with the minimal TLH.

By cutting off the solutions dominated by *mTTPT* or *mTLH* before non-dominated sorting, time complexity can be decreased greatly. The methods to find out the solutions dominated by *mTTPT* and *mTLH* are described in Theorems 5 and 6 respectively.

#### **Theorem 5**

*If a solution’s TLH is more than mTTPT’s, then the solution must be dominated by mTTPT.*

#### *Proof*

See Proof of Theorem 5 in “Appendix”.

#### **Theorem 6**

*If a solution’s TTPT is more than mTLH’s, then the solution must be dominated by mTLH.*

#### *Proof*

See Proof of Theorem 6 in “Appendix”.

The improved exact algorithm by decreasing time complexity is described as follows. 
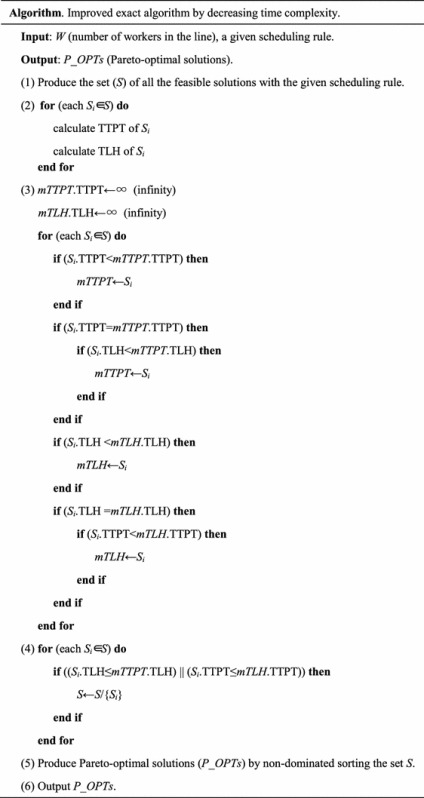


In step (1), both time complexity and space complexity are *O*(*N*).

In step (2), time complexity is *O*(*N*) and space complexity is *O*(4*N*).

Step (3) is to obtain *mTTPT* and *mTLH* by traversal all the feasible solutions. Both time complexity and space complexity are *O*(*N*).

Step (4) is to obtain the solutions non-dominated (assume the number is *K*) by *mTTPT* or *mTLH* by traversing all the feasible solutions. Both time complexity and space complexity are *O*(*N*).

Step (5) is to obtain the exact Pareto-optimal solutions by non-dominated sorting the *K* solutions obtained in step (4). The time complexity is *O*(2*K*^2^) and space complexity is *O*(*K*).

The key of the improved exact algorithm by decreasing time complexity is leis in step (4), i.e., the operation of “*S*←*S*/{*S*_*i*_}”. That cuts off the solutions dominated by *mTTPT* or *mTLH* before non-dominated sorting, i.e., step (5).

Obviously, for the improved exact algorithm by decreasing time complexity, the time complexity is the maximum between *O*(2*K*^2^) and *O*(*N*). Space complexity is *O*(4*N*) still. Therefore, we propose another improved exact algorithm by decreasing space complexity.

### The improved exact algorithm by decreasing space complexity

If we partition all the feasible solutions (*N*) in several sub-sets to obtain the non-dominated solutions of each sub-set, then the space complexity will be decreased. Subsequently, the Pareto-optimal solutions can be obtained by sorting the non-dominated solutions in all sub-sets. The improved exact algorithm by decreasing space complexity is described as follows.
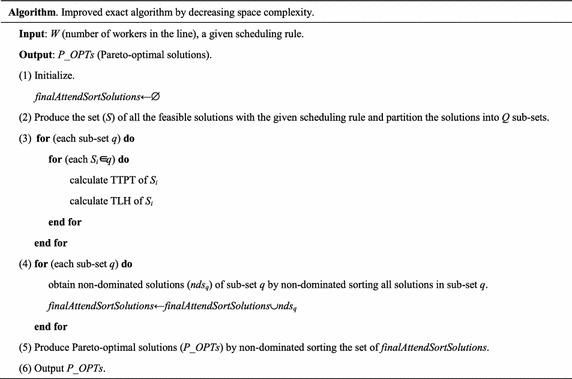


Step (1) is to initialize.

Step (2) is to partition all the produced feasible solutions into *Q* sub-sets. In each sub-set, there are approximately $\frac{N}{Q}$ solutions.

Step (3) calculates the TTPT and TLH of each solution in each sub-set. The time complexity is $O\left( {\frac{N}{Q}} \right)$ and space complexity is $O\left( {4\frac{N}{Q}} \right)$.

Step (4) obtains non-dominated solutions (assume the number is *S*_*q*_) of sub-set *q* using non-dominated sorting. The time complexity is $O\left( {2\left( {\frac{N}{Q}} \right)^{2} } \right)$ and space complexity is $O\left( {\frac{N}{Q}} \right)$.

Step (5) obtains the exact Pareto-optimal solutions by non-dominated sorting the $\sum\nolimits_{q = 1}^{Q} {S_{q} }$ non-dominated solutions, where $\sum\nolimits_{q = 1}^{Q} {S_{q} }$ non-dominated solutions refer to all sub-sets’ non-dominated solutions obtained in step (4). Time complexity is $O\left( {2\left( {\sum\nolimits_{q = 1}^{Q} {S_{q} } } \right)}^{2} \right)$ and space complexity is $O\left( {\sum\nolimits_{q = 1}^{Q} {S_{q} } } \right)$.

The key of the improved exact algorithm by decreasing space complexity lies in steps (2) and (4). Step (2) partitions the whole solution space into several sub-spaces. Step (4) produces the solutions to attend the final non-dominated sorting by aggregating the non-dominated solutions of all sub-spaces.

For the improved exact algorithm by decreasing space complexity, space complexity is the maximum between $0\left( {4\frac{N}{Q}} \right)$ and $O\left( {\sum\nolimits_{q = 1}^{Q} {S_{q} } } \right)$, and time complexity is the maximum between $O\left( {2\left( {\frac{N}{Q}} \right)^{2} } \right)$ and $O\left( 2{\left( {\sum\nolimits_{q = 1}^{Q} {S_{q} } } \right)^{2} } \right)$.

## Computation experiments

### Test instances

Tables 5, 6, 7, 8 and 9 show the parameters, data distribution and detail data of level of skill of workers, coefficient of influencing level of skill to multiple stations for workers and data of batches used in test, respectively. From the 5 Tables, it can be observed that the lot size of each batch is *N*(50,5) and the ability of workers is also different with stations and *N*(0.2,0.05). The detailed data of *ε*_*i*_ and batches are given in Tables 7 and 8 respectively.

**Table 5:** Parameters in the experiments

Product types	Batch size	*ε*_*i*_	*SL*_*n*_	*SCP*_*n*_	*T*_*n*_	*η*_*i*_
5	*N*(50,5)	*N*(0.2,0.05)	2.2	1.0	1.8	10

*N*(50,5): Normal distribution (μ = 50, σ  = 5)

**Table 6:** Data distribution of worker’s level of skill (*β*_*ni*_)

Product type				
1	2	3	4	5
*N*(1,0.1)	*N*(1.05,0.1)	*N*(1.1,0.1)	*N*(1.15,0.1)	*N*(1.2,0.1)

**Table 7:** Data of worker’s level of skill (*β*_*ni*_)

Worker\product	1	2	3	4	5
1	0.92	0.96	1.04	1.09	1.2
2	0.95	0.97	1.09	1.12	1.18
3	0.99	1.01	1.05	1.09	1.21
4	1.03	1.07	1.09	1.12	1.25
5	0.96	1.02	1.05	1.1	1.18
6	1.01	1.1	1.1	1.15	1.23
7	1.04	1.07	1.09	1.17	1.24
8	0.98	1.02	1.1	1.11	1.2
9	0.97	1.03	1.12	1.19	1.26
10	0.98	1.06	1.13	1.18	1.28

**Table 8:** Coefficient of influencing level of skill to multiple stations for workers (*ε*_*i*_)

Worker	1	2	3	4	5	6	7	8	9	10
*ε*_*i*_	0.18	0.19	0.2	0.21	0.2	0.2	0.2	0.22	0.19	0.19

**Table 9:** Data of batches

Batch number	1	2	3	4	5	6	7	8	9	10	11	12	13	14	15
Product type	3	5	3	4	1	4	1	2	2	3	2	4	3	4	5
Batch size (*B*_*m*_)	55	53	54	49	49	55	54	48	48	48	46	58	48	52	48
Batch number	16	17	18	19	20	21	22	23	24	25	26	27	28	29	30
Product type	5	1	4	2	5	1	3	4	5	2	3	1	4	2	3
Batch size (*B*_*m*_)	51	54	57	54	49	53	46	45	46	45	44	53	47	53	52

Table 5 shows that the mean of skill level of each worker for product type *n* (*β*_*ni*_) ranges from 1 to 1.2 and the standard deviations are fixed to 0.1. The detailed data of *β*_*ni*_ are given in Table 6.

For the instance with *W* workers, we use the following data set from Tables 5, 6, 7, 8, 9: the entire Table 5, the first *W* rows of Tables 7 and 8, and the entire Table 9.

### Hardware and software specifications

The two improved exact algorithms were coded in C# and executed on an Intel Core(TM) i7-4790 CPU @ 3.6 GHz under Windows 7 using 8 GB of RAM.

### Computation results of the improved exact algorithm by decreasing time complexity

**Fig. 14:**
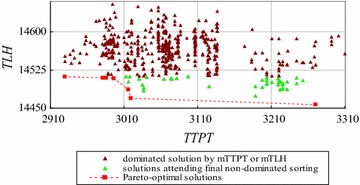
The solutions dominated by *mTTPT* and *mTLH* for the instance with 5 workers

**Fig. 15:**
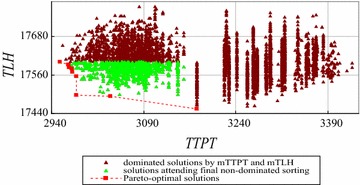
The solutions dominated by *mTTPT* and *mTLH* for the instance with 6 workers

**Fig. 16:**
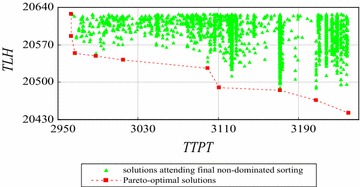
The solutions to attend non-dominated sorting for the instance with 7 workers

The enumeration based on non-dominated sorting cannot solve the instances with more than 6 workers using a *SRRSS*, such as FCFS. We use the improved exact algorithm by decreasing time complexity to solve the instances with 5, 6 and 7 workers. The computation results are shown in Figs. 14, 15, 16 respectively.

Figure 14 shows 7 Pareto-optimal solutions, 541 feasible solutions, 493 solutions dominated by *mTTPT* or *mTLH*, and left 48 solutions to attend non-dominated sorting for the instance with 5 workers. Similarly, Fig. 15 shows 9 Pareto-optimal solutions, 4683 feasible solutions, 3831 solutions dominated by *mTTPT* or *mTLH*, and only left 852 solutions to attend non-dominated sorting for the instance with 6 workers. Figure 16 shows the Pareto-optimal solutions of the instance with 7 workers, where 10 Pareto-optimal solution and 2437 solutions to attend non-dominated sorting.

Table 10 shows the performance of the improved exact algorithm by decreasing time complexity for different instances.

**Table 10:** Performance of the improved exact algorithm by decreasing time complexity

Number of workers (W)	5	6	7	8
Number of all solutions (N)	541	4683	47,293	545,835
Left solutions (K)	48	852	2437	9749
Solutions cut off	493	3831	44,856	536,086
Ratio of solutions cut off (%)	91	82	95	98.2
Time of enumeration based-NS (second)	0.06	1.4	–	–
Time of NS in step (5) (second)	0.002	0.03	0.4	–
Total time of the improved algorithm(second)	0.035	0.28	2.87	–

From Table 10, we can see that the improved exact algorithm by decreasing time complexity has a better performance than the enumeration based on non-dominated sorting because of cutting off approximately 89 % non Pareto-optimal solutions before running non-dominated sorting. Compared with the original non-dominated sorting algorithm, the step (5) saves approximately 98 % computational time. For example, for the instances with 5 and 6 workers, in step (5) the saved computational time are $\left( {1 - \frac{0.002}{0.06}} \right)*100\;\% = 97\;\%$ and $\left( {1 - \frac{0.03}{1.4}} \right)*100\;\% = 98\;\%$ respectively. That is because, by cutting off non-dominated solutions, the time complexities of non-dominated sorting are improved by $\left( {\frac{48}{541}} \right)^{2} *100\;\%$ for the instance with 5 workers and by $97\;\% = \left( {1 - \left( {\frac{852}{4,683}} \right)^{2} } \right)*100\;\%$ for the instance with 6 workers.

The enumeration based on non-dominated sorting cannot solve the instances with more than 6 workers. The improved exact algorithm by decreasing time complexity solves the instance with 7 workers in 2.87 s. The time complexities of non-dominated sorting are improved by $99.7\;\% = \left( {1 - \left( {\frac{2,437}{47,293}} \right)^{2} } \right)*100\;\%$.

Moreover, we can easily observe the computation time of enumeration based on non-dominated sorting increases exponentially with the number of all solutions, however, the total time of the improved exact algorithm by decreasing time complexity increases linearly with the number of all solutions.

The improved exact algorithm by decreasing time complexity cannot solve the instance with more than 7 workers because 9749 left solutions cannot be solved by non-dominated sorting.

### Computation results of the improved exact algorithm by decreasing space complexity

**Fig. 17:**
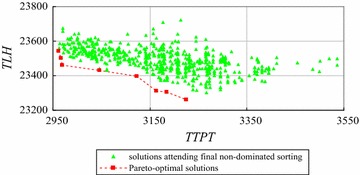
The Pareto-optimal solutions of the instance with 8 workers

**Fig. 18:**
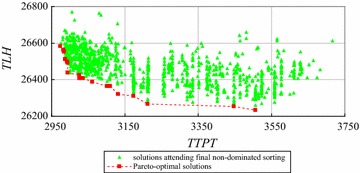
The Pareto-optimal solutions of the instance with 9 workers

We use the improved exact algorithm by decreasing space complexity to solve the instances with 8 and 9 workers using FCFS rule. The numbers of sub-sets (*Q*) of instances with 8 and 9 workers are set as 8 and 9 respectively. The computation results are shown in Figs. 17 and 18 respectively.

Figure 17 shows that there are final 599 solutions to attend non-dominated sorting and 8 Pareto-optimal solutions for the instance with 8 workers. Similarly, Fig. 18 shows there are 19 Pareto-optimal solutions generated by non-dominated sorting final 1142 solutions in all 7,087,261 feasible solutions for the instance with 9 workers.

Table 11 shows the performance of the improved exact algorithm by decreasing space complexity for different instances.

**Table 11:** Performance of the improved exact algorithm by decreasing space complexity

Number of workers (W)	5	6	7	8	9
Number of all solutions (N)	541	4683	47,293	545,835	7,087,261
Left solutions $\left(\sum\limits_{q = 1}^{Q} {S_{q} }\right)$	49	133	249	599	1142
Solutions cut off	492	4550	47,044	545,236	7,086,119
Ratio of solutions cut off (%)	91	97	99.5	99.9	99.98
Time of enumeration based-NS (second)	0.06	1.4	–	–	–
Total time of the improved algorithm (second)	0.03	0.27	2.24	25.9	342

From Table 11, we can see that the improved exact algorithm by decreasing space complexity has a better performance than the improved exact algorithm by decreasing time complexity. That is because the improved exact algorithm by decreasing space complexity cuts off more non Pareto-optimal solutions before running final non-dominated sorting (i.e., Tables 10, 11). Moreover, the total time of the improved exact algorithm by decreasing space complexity increases linearly with the number of all solutions (*N*).

However when producing all the feasible solution (*N*) is not possible, the improved exact algorithm by decreasing space complexity cannot obtain the Pareto-optimal solutions. For example of the instance with 10 workers using FCFS rule, there are 102,247,563 feasible solutions of line-*seru* conversion, and the computer cannot produce all the feasible solutions.

## Conclusions

Our contributions in this paper are summarized as following. First, we investigate the significant influence of the 10 selected scheduling rules on the TTPT and TLH performances of *seru* system. Subsequently, we clarify the complexities of *seru* load and line-*seru* conversion for ten different scheduling rules in detail. Second, we develop two improved exact algorithms based on reducing time complexity and space complexity respectively, to obtain Pareto-optimal solutions of multi-objective line-*seru* conversion. Compared with the enumeration based on non-dominated sorting, the two proposed algorithms greatly decrease time complexity and space complexity respectively and improve the computation performance by approximately 98 %.

The line-*seru* conversion is a real problem in Japan electronics industry, therefore there are still a lot of works should be performed. For example, the influence of scheduling rules on the performance improvements in line-*seru* conversion should be further researched. Furthermore, other important production performances of *seru* system should be evaluated, such as balancing (Esmaeilbeigi et al. 2015) and WIP. In addition, the situations under which workers can’t operate all tasks in a *seru* should be investigated, i.e., the fundamental principles of hybrid *seru* system with a short line and operation management of the *seru* system including divisional *serus*. Moreover, the further research should consider the number of assembly tasks varying with the product types. Also, the optimal methods to train the multi-skilled workers in *seru* production should be studied in future.

## Appendix: Proofs of Theorems

### Proof of Theorem 1

Without given a scheduling rule, in *seru* load, each product batch (*M*) can be assigned into any *seru* (*J*) of the given *seru* formation.

### Proof of Theorem 2

Given a *SRRSS*, if *M* (number of batches) ≥ *J* (number of *serus*), then the first *J* batches are assigned to the *J**serus* according to the *seru* sequence and the *SRRSS*. That is to say a *seru* sequence of given *seru* formation produce an allocation result for the first *J* batches. Subsequently, the allocation result of the last *M* − *J* batches can be obtained based on the result of the first *J* batches. This is because batch *J* + 1 will be assigned to the *seru* with the earliest completion time, batch *J* + 2 will be assigned to the *seru* with the earliest completion time, and so on. Therefore, using a *SRRSS* each *seru* sequence of the given *seru* formation produces a *seru* load result. For a *seru* formation with *J**serus*, there are *J*! *seru* sequence. Thus, if *M* ≥ *J*, the number of solutions of *seru* load (*S*) equals *J*! = $P_{J}^{J}$.

### Proof of Theorem 3

Given a *SRRSS*, if *M* < *J*, only the first *M serus* are used to assemble the *M* batches. There are $C_{J}^{M}$ solutions of selecting arbitrary *M**serus* from the *J**serus*. Thus, if *M* < *J,* the number of solutions of *seru* load (*S*) equals $C_{J}^{M} P_{M}^{M} = P_{J}^{M}$.

### Proof of Theorem 4

Consider {*S*_1_,*S*_2_,…,*S*_*J*_} is the *seru* set and {*B*_1_,*B*_2_,…,*B*_*M*_} is the batch set. If a *SRUSS* is used in *seru* load, then the *seru* sequence does not influence the scheduling results of *seru* load. Regardless of the *seru* sequence the first selected batch *m* (i.e., *B*_*m*_) is always assigned to *seru**j* (i.e., *S*_*j*_) according to the *SRUSS*, the second selected batch is always assigned to the corresponding *seru*, and so on. Thus, given a *seru* formation the result of *seru* load with *SRUSS* is only.

### Proof of Theorem 5

According to Definition 1, any solution’s TTPT is not less than *mTTPT’s*. If a solution’s TLH is more than *mTTPT’s*, then it must be dominated by *mTTPT*.

### Proof of Theorem 6

According to Definition 2, any solution’s TLH is not less than *mTLH’s*. If a solution’s TTPT is more than *mTLH’s*, then it must be dominated by *mTLH*.
